# Ethyl Acetate Extract of Licorice Root *(Glycyrrhiza glabra)* Enhances Proliferation and Osteogenic Differentiation of Human Bone Marrow Mesenchymal Stem Cells

**Published:** 2018

**Authors:** Arezou Azizsoltani, Khosro Piri, Sahar Behzad, Masoud Soleimani, Mina Nekouei, Zahra Mahmoudi, Asad Kazemi

**Affiliations:** a *Department of Biotechnology, Faculty of Agriculture, Bu-Ali Sina University, Hamedan, Iran. *; b *Department of Medical Biotechnology, Faculty of Advanced Sciences, Tabriz University of Medical Sciences, Tabriz, Iran.*; c *Department of Pharmacognosy, School of Pharmacy, Shahid Beheshti University of Medical Sciences, Tehran, Iran. *; d *Evidence-based Phytotherapy and Complementary Medicine Research Center, Alborz University of Medical Sciences, Karaj, Iran.*; e *Department of Hematology, Faculty of Medical Sciences, Tarbiat Modares University, Tehran, Iran. *; f *Department of Phytochemistry, Medicinal Plants and Drug Research Institute, Shahid Beheshti University, Tehran, Iran. *; g *Department of Life Science Engineering, Faculty of New Science and Technologies, University of Tehran, Tehran, Iran.*; h *Department of Biology, Faculty of Sciences, University of Mohaghegh Ardabili, Ardabil, Iran.*

**Keywords:** *Glycyrrhiza glabra*, Fabaceae, Phytoestrogen, Osteoporosis, Mesenchymal stem cell, Differentiation

## Abstract

*Glycyrrhiza glabra (G. glabra)* has been used as a flavoring and sweetener agent, in addition to its therapeutic properties. It is rich in phytoestrogen and may prevent osteoporosis caused by estrogen deficiency; however, there is no evidence for its effects on proliferation and osteogenesis in mesenchymal stem cells. So, we were encouraged to investigate whether the ethyl acetate extract of licorice root as a source of phytoestrogen can act similar to estrogen in cell culture. Furthermore, the analysis of the licorice extract (LE) based on HPLC-DAD-ESI-MS indicated that LE comprises phytoestrogen compounds, such as glabridin and glabrene. In this study, the effects of LE on proliferation of human bone-marrow mesenchymal stem cells (hBM-MSCs) were investigated using MTT assay. In addition, its effects on the osteogenesis were evaluated using alkaline phosphatase activity (ALP), calcium deposition, and bone specific gene expression such as ALP, osteocalcin, Runx2, and BMP-2. The quantitative gene expression was studied by real-time RT-PCR. Our results showed a significant increase in proliferation in presence of LE in concentration 10-50 µg/mL. The differentiation of hBM-MSCs increased in doses of LE (10-25 µg/mL) compared to the control group. The effects of LE were similar to those of 17β-estradiol (E2) (10^-8^ M) and were abolished by ICI 182,780 an antagonist of estrogen receptor (ER) (10^-7^), indicating that the stimulatory effects of LE occur through estrogen receptor-mediated mechanism . Taking these into account, LE may be a potential candidate for prevention of osteoporosis in menopausal women.

## Introduction

Osteoporosis is a common disease of bone in elderly people, which causes a reduction in bone density and consequently increased risk of fracture. Osteoporosis is traced back to an imbalance of osteoclastic bone resorption *vs.* osteoblastic bone formation in the bone remodeling process (1). Estrogen is known as an important regulator in bone remodeling playing a significant role in the differentiation of osteoblast progenitor cells into mature osteocytes. In women, age-related decline in estrogen following menopause can lead to a reduction in bone mineral density (2). The most common remedy for osteoporosis is hormone replacement therapy (HRT) which is not recommended according to world health organization (WHO) investigations (3), because HRT is associated with increased risk of breast, endometrial and ovarian cancers and cardiovascular disease (4-6). Recently, most studies have tended to discover alternate treatments such as natural compound application (7). Phytoestrogens are natural substances that mimic estrogen and can replace hormone therapy to inhibit menopausal symptoms and osteoporosis (8). These substances contain lignans and flavonoids (isoflavones, and coumestans) which are known as selective estrogen receptor modulators (SERM) due to acting as antagonists of estrogen in breast tissue and agonists in bone (9). *Glycyrrhiza glabra* which is known as licorice belonging to the Fabaceae, is native to southern Europe and parts of Asia including Iran (10). Licorice roots have been used as a remedy for cough, laxative, menopausal hot flashes, peptic ulcer, and viral diseases (11-13). Besides, the prevention of LE on proliferation of breast, colon and prostate cancer cell-line and also diverse pharmacological effects such as antimicrobial, antioxidant, and anti-inflammatory activity were investigated (14-17). There are several compounds in LE which have phytoestrogen properties such as hispaglabridin A, hispaglabridin B, licochalcon-A, licochalcon-B and formononetin and phyto-SERM such as glabridin and glabrene (18, 19). Simons *et al.* showed that the ethyl acetate extract of licorice root displayed estrogenic activity in yeast estrogen bioassay; so we chose the ethyl acetate extract of licorice root (20). Although the stimulatory effect of glabridin and glabrene were examined on bone metabolism and was similar to E2, the mechanism of action an licorice extract on osteogenic differentiation and proliferation of mesenchymal stem cells have not been evaluated (21-23). BM-MSCs can be differentiated into multiple lineages such as osteoblasts, chondrocytes, and adipocytes (24). To screen the phytochemical compounds in our study, we applied HPLC-DAD-MS analysis and then investigated the ability of Ethyl-acetate extract of licorice root on proliferation and osteoblastic differentiation of hBM-MSCs. Moreover, we used ICI 182,780, a specific estrogen receptor alpha (ERα) antagonist to determine whether licorice extract could act through ER-dependent mechanism (25).

## Experimental

*Plant extract*

The root and stolon of the licorice was collected in autumn at Hamadan, Iran and were identified by the department of botany (Bu-Ali Sina University). A voucher specimen of this plant was deposited at the Bu-Ali Sina University herbarium (BASU) for future reference. The air-dried and powdered root of licorice was macerated in Hexane (1:4) and shaked for 24 h at room temperature to remove lipids. Then, the residues were extracted with ethyl acetate. The extract was concentrated with a rotary evaporator at temperature less than 40 °C and then freeze dried (yielding 12.3% of dry residue). The extract was dissolved in dimethyl sulfoxide (DMSO) at a final concentration of 500 µg/mL and filtered through a Millipore filter (0.22 µm). The stock solution was serially diluted with the medium. For all experiments final concentration of DMSO was less than 0.1%.

*HPLC-DAD-ESI-MS analysis*

HPLC-DAD-ESI-MS was carried on an Agilent HPLC system (Waldbronn, Germany) equipped with a binary pump, a diode array detector and coupled to a Finnigan ^TM^ LCQ ^TM^ DECA instrument equipped with an ion trap mass spectrometer and an electrospray ionization (ESI) source (electrospray voltage: 4.5 kV, sheath gas and auxiliary gas: nitrogen, capillary temperature: 200 °C). RPLC analysis was carried out on a Macherey-Nagel Nucleodur C18ec (5 µm, 4.6 × 250 mm, Germany) column. Mobile phase composed of water (with 0.1% formic acid, A) and acetonitrile (with 0.1% formic acid, B). The gradient elution was from 45% B to 60% B within 50 min, then isocratically for further 10 min and after that increased to 80% in 20 min. the flow rate was 0.5 mL/min. HPLC chromatograms were recorded at 254 nm, 283 nm, and 330 nm. The mass spectrometer data were collected over a *m/z* range of 150-2000 in both negative ion (NI) and positive ion (PI) modes. Data dependent MS^2^ analysis was performed with collision energy of 35%. Data acquisition and processing were performed with the Xcalibur 2.0 SR2 software (Thermo Scientific).

**Table 1:** Compounds assigned in the *G. glabra* extract by LC-MS.

**No**	**Rt. (min)**	**UV (nm)**	**Identification**	**[M-H]** ^-^	**MS** ^2 ^ **product ions (relative intensity)**	**[M+H]** ^+^
1	13.6	230, 280	Dihydroxy-glabridin	357	135, 235, 339	341
2	17	230, 280, 340	Hydroxy-glabrene	337	161, 268, 293, 309, 322	339
3	18	220,280	Hydroxy-4’-O-methylglabridin	353	165, 201, 321, 309, 338	355
4	19.8	217, 250, 285, 325	Glabrene	321	175, 293, 306	323
5	25.5	280	Hydroxy-glabridin	339	135,203,221,321	341
6	26	230, 280	Glabridin	323	121, 135, 201	325
7	27	222, 250, 302	Glabrone	335	213, 291, 292, 307, 320	337
8	36	230, 280, 290	Hispaglabridin A	391	335	393
9	46	230, 280, 320	Hispaglabridin B	389	373	391

**Figure 1:**
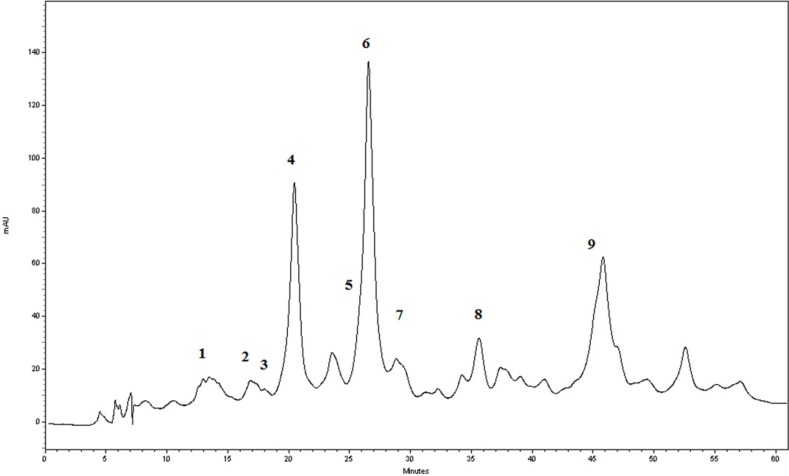
The RP-HPLC-DAD chromatogram of the *G. glabra* extract at 283 nm

**Figure 2:**
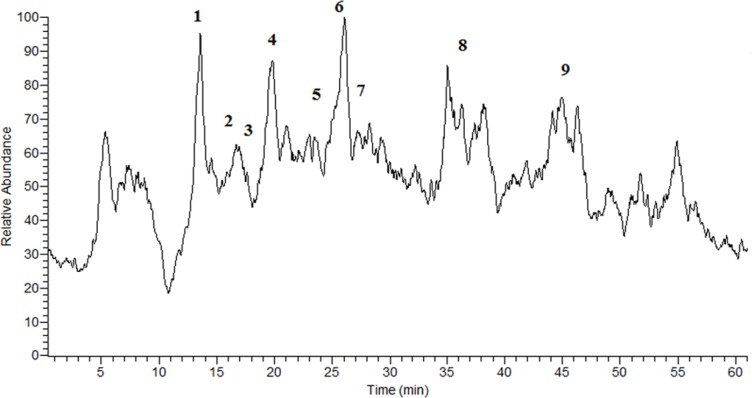
The TIC chromatogram of the *G. glabra* extract obtained in NI mode.

**Figure 3:**
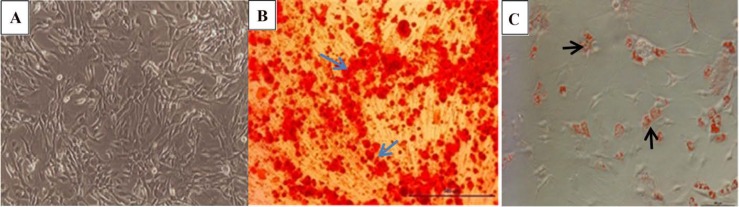
(A) Morphology of undifferentiated Mesenchymal Stem Cells. (B) Osteogenic differentiation of MSCs was visualized by Alizarin Red which stained calcium deposited in extracellular matrix . (C) adipogenic differentiation was confirmed by lipid vacuoles stained with Oil Red staining. (Magnification 10X).

**Figure 4:**
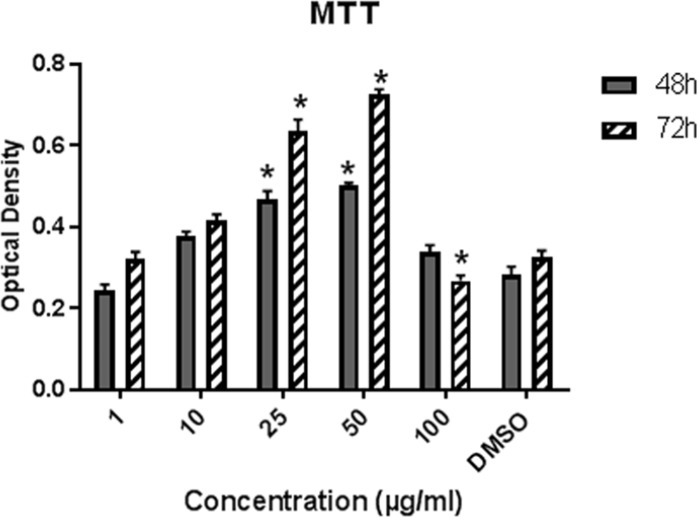
Effect of LE on the proliferation of hBM-MSCs. Cells were exposed to LE (1-100 µg/mL) and 0.1% DMSO for 48 and 72 h. Data represent mean ± SD and expressed as an Optical density. ^*^P ˂ 0.05 significantly different compared to control (n = 4).

**Figure 5:**
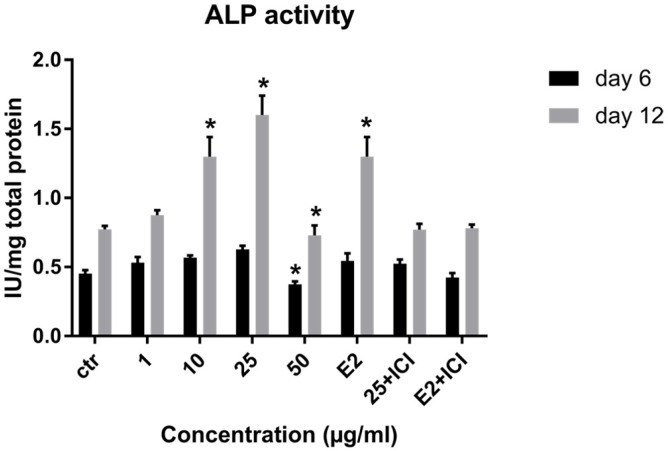
Effect of LE on Alkaline phosphatase activity of hBM-MSCs. Data shown are mean ± SD. ^*^P ˂ 0.05 *vs.* control (n = 5).

**Figure 6:**
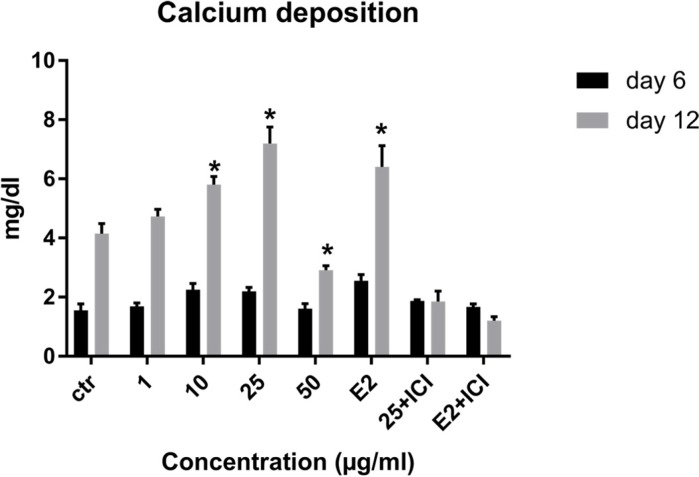
Effects of LE on calcium deposition of MSCs. Data shown are mean ± SD. ^*^P ˂ 0.05 *vs.* control.

**Figure 7:**
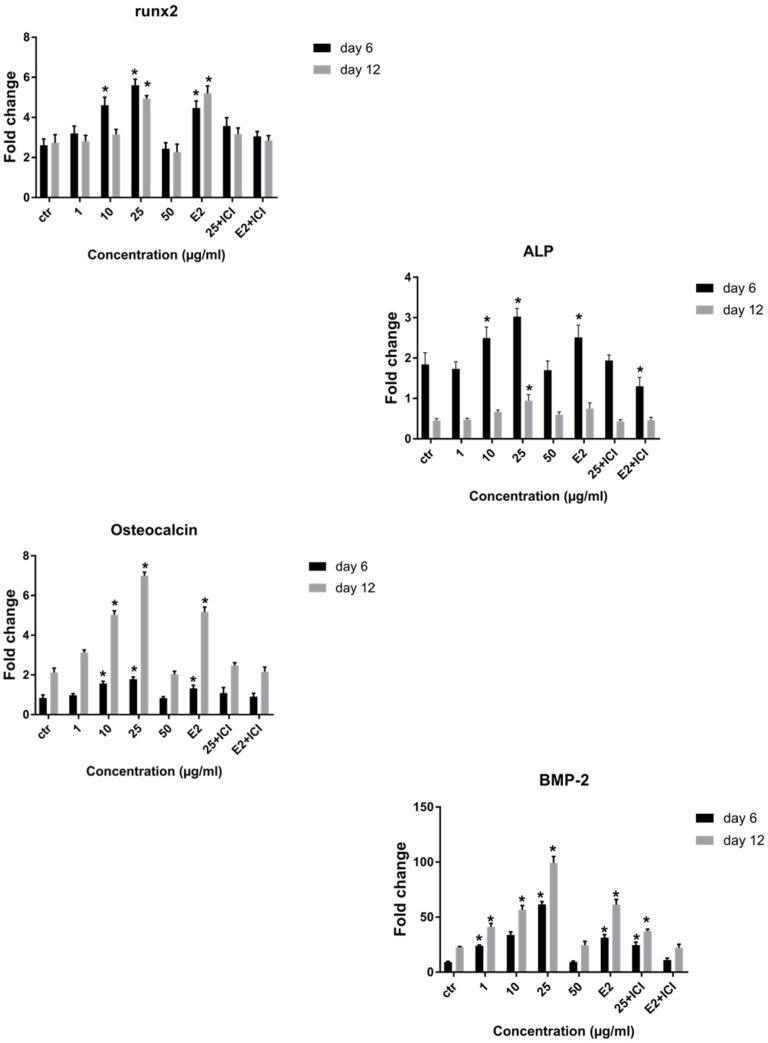
The real time RT-PCR analysis of osteoblastic genes. Changes in expression of Runx2, ALP, BMP-2 and osteocalcin were analyzed in cells, which were grown in osteogenic medium (control) plus different doses of LE or E2 with or without ICI 182,780 on day 6 and 12. Data represent mean ± SD expressed as a percentage of control. ^*^P ˂ 0.05 significantly different compared to control (n = 4).

*Cell culture*

The human bone marrow mesenchymal stem cells were gifted by Dr Soleimani and were isolated as described previously (Yazdani *et al*., 2013). The cells were grown in DMEM-LG (Gibco®, grand island, NY, USA) supplemented with 10% fetal bovine serum (FBS) (Gibco®), 2 mM L-glutamin (Gibco®), Penicillin (100 U/mL, Gibco®) and streptomycin (100 µg/mL, Gibco®) and incubated at 37 °C under 5% CO_2_ atmosphere. The medium was changed twice a week. When the adherent cells reached confluence (P_0_) they were trypsinized by 1 mL of 0.05% trypsin (Sigma-Aldrich®, USA) and subcultured into new flasks. For most of the experiments, we used the hBM-MSCs at the 3^rd^ passages.

*Differentiation and Flow cytometry analysis of hBM-MSCs*

For osteogenesis assay, the cells were grown in osteogenic induction medium consisting of (DMEM+10%FBS) supplemented with 10 nM Dexamethasone, 0.2 mM ascorbic acid 2-phosphate, and 10 mM β-glycerol phosphate (all from Sigma-Aldrich®, St. Louis, USA) and the medium was changed twice per week for up to 21 days. The cells were fixed with paraformaldehyde for 20 min at 4 °C and stained with Alizarin Red 2% at pH 7.2. For adipogenesis the cells were cultured in adipogenic medium, including DMEM supplemented with 10% FBS, indomethacin (50 µg/mL), dexamethasone (1 µM), and ascorbate-2-phosphate (50 µg/mL). The culture medium was changed tow times per week for up to 21 days. The cells were fixed in methanol for 45 min and visualized with Oil Red staining (all substances from *Sigma-Aldrich*®). The cells were observed under LB290 inverted microscope (Labomed Inc., USA) with magnification 10X. The isolated cells were characterized using flow cytometry, which analyzed the expression surface markers such as CD31, CD44, and CD73. The cells were detached using trypsin/EDTA and incubated with the specific antibodies or isotype control antibodies, in 100 µL 3% BSA/PBS for 1h at 4 °C. Then, the cells were fixed with 1% paraformaldehyde and analyzed using flow cytometry (BD FACSCalibur™) and FlowJo® software (Tree Star, Ashland, USA).

*MTT assay*

The effect of different concentration of LE on the proliferation rate of hMSCs was evaluated via MTT assay (26). The cells were plated at the density of 10^4^ cell/well in 96-well culture plate. After 24 h incubation, the cells were treated with medium plus different doses of LE (0.1, 10, 25, 50 and 100 µg/mL) and 0.1% DMSO as the control group. Cells were incubated at 37 °C for 48 and 72 h and then 20 µL of MTT solution (5 mg/mL in DMEM) was added to each well and incubated for 4 h at 37 °C. The supernatant was discarded and 200 µL DMSO was added to dissolve the formazan salt. The optical density was measured at 570-630 nm

*ALP activity*

To study the ALP activity, hMSCs were seeded in 24-well plates at a density of 10^5^ cells/well and after 24 h cells were exposed to test agent (osteogenic medium (OS) + LE, OS + 0.1% DMSO, OS + E_2_). After 6 and 12 days of treatment total protein of cells was extracted using cell lysis buffer. The cells were washed twice with PBS and lyzed with RIPA and centrifuged at 14000×g for 5 min. Aliquots of supernatant were used for ALP activity. Enzyme activity was measured with the ALP assay kit according to the manufacturer’s instructions (Parsazmun, Tehran, Iran) using ρ-nitrophenyl phosphate as substrate. ALP activity was normalized according to the total protein.

*Measurement of calcium content*

To analyze the amount of calcium deposition in our study, cultures were decalcified with 0.6 N Hcl (Merck), previously described (27). Calcification was evaluated by calcium content assay kit (Parsazmun,Tehran,Iran) using the cresophthalein compelexon method. The absorbance at 570 nm was measured. The calcium deposited of culture was normalized to serial dilution of various concentrations of calcium versus optical density (OD).

*Real-time RT PCR*

The expression of osteogenic mRNA was evaluated using real-time RT-PCR (27). The cells were cultured in 6-well plate and after 24 h exposed to experimental treatment. The medium was replaced every 3 days. After 6 and 12 days total RNA was isolated by RNeasy kit according to manufacturer’s instructions (Qiagen, Germantown, MD, USA). cDNA was synthesized by revert Aid first strand cDNA synthesis kit (Fermentase, Burlington, Canada). Amplification was performed by Rotor-Gene Q real-time analyzer (Corbett, Australia) and SYBR Premix Ex Taq (Takara Bio Inc., Japan). Our results were analyzed by Rotor-Gene Q Software (Corbett) and melting curve analysis was used to confirm PCR specificity and threshold cycle average. The primer sequences are emerged in Table S1 (Supplementary File). Fold change was calculated according to ΔΔCT method. GAPDH was used as internal control and target genes were calibrated to MSC P3. Two-step amplification protocol was chosen, consisting of initial denaturation at 95 °C for 3 min followed by 40 cycles with 5 sec denaturation at 95 °C and 20 sec annealing/extension at 60 °C. Each reaction was duplicated and repeated twice. 

*Osteogenic analysis after the addition of ICI 182,780*

The MSCs were plated in 6-well plate, after incubation for 24 h, the medium changed into osteogenic medium with LE (25 µg/mL) or E2 in the presence or absence ICI182,780 (Cayman chemical, USA). After 6 and 12 of treatment, the cells were collected for ALP activity, calcium deposition, and real-time PCR.

*Statistical analysis*

The results are expressed as mean ± SD. Significant levels among different groups were analyzed using GraphPad Prism 7.01, Two-way ANOVA. *P*-value < 0.05 was regarded as statistically significant.

## Results

*Identification of Compounds in the G. glabra extract*

In order to identification of some phytochemical compounds in the ethyl acetate extract of *G. glabra* root, HPLC-ESIMS analysis was applied in both positive and negative ion modes. As a preliminary experiment, the peak of glabridin in the extract was assigned by comparing its retention time, UV spectra, as well as parent and production ions to the standard. The RP-HPLC-DAD profile of the extract (Figure 1) and the total ion current (TIC) chromatogram obtained in the NI mode are displayed (Figure 2). The compounds were numbered according to the peak elution order in the chromatogram as shown in Table 1.

*Characterization of BM-MSCs*

To characterize isolated mesenchymal stem cells, we used the osteogenesis and the adipogenesis potential of these cells and the expression of mesenchymal-related surface marker. Figure 3A shows the characteristic spindle shape and plastic adherent properties of MSCs. In Figure 3B, red spots illustrate calcium nodules and mineralization in MSCs which confirmed undergoing osteogenic differentiation and in Figure 3C. Lipid droplets in MSCs which were cultured in appropriate medium exhibited adipogenic differentiation potential. In addition, flow cytometry analysis showed that MSCs passage 2 cells were positive for CD44, CD73 and were negative for CD31 which confirmed their non-hematopoietic origin (Figure S1, Supplementary File). 

*Effect of licorice extract on the proliferation of hBM-MSCs*

The proliferation of BM-MSCs in various concentrations of LE (1-100 µg/mL) was evaluated via MTT after 48 and 72 h of treatment (Figure 4). LE significantly increased cell proliferation in concentrations 10, 25, and 50 µg/mL after 48 h of treatment as compared to control (*P* ˂ 0.05) and a higher increase was achieved after 72 h exposure to 50 µg/mL of plant extract. The proliferation of cells was inhibited in a 100 µg/mL concentration as this concentration exhibited toxic effects on cell proliferation.

*Alkaline phosphatase activity assay*

We evaluated the quantitative ALP activity as a biochemical marker of osteogenesis in BM-MSC in the presence or absence (control) of LE and E2 on day 6 and 12 of treatment. LE (10-25 µg/mL) caused a significant enhancement in the enzyme activity of stem cells on day 6 and 12 of the treatment. As shown in Figure 5 the maximum effect of LE was observed in 25 µg/mL on day 12 compared to the control.

*Effect of LE on calcium deposition*

Calcium deposition as a late marker of osteogenesis was measured to assess the effect of LE on osteoblastic differentiation of MSCs on days 6 and 12. As observed from the results, the amount of calcium deposition increased over time in all treatments and a significant increase was observed on day 12 at concentration of 10-25 µg/mL. The maximum effect was at the concentration of 25 µg/mL (Figure 6).

*Effect of LE on mRNA expression of bone-related genes*

Quantitative real-time PCR was utilized to study the effect of LE on the expression of osteoblast specific genes, including Runx2, ALP, osteocalcin, and BMP2 on day 6 and 12 of osteogenic culture. RT-PCR analysis showed that Runx2 and ALP mRNA were up-regulated on day 6 followed by a decrease on day 12 and had a 2-fold and 2.4-fold increase respectively, compared to control at concentration of 25 µg/mL. Results showed a significant increase in osteocalcin gene expression in the presence of LE on days 6 and 12 and reached a peak at concentration of 25 µg/mL. Notably BMP2 gene expression increased and had a 4.5-fold increase compared to control and a greater level of BMP2 expression was achieved on day 12 of culture (Figure 7). We observed in gene expression pattern that E2 (10^-8^M) group is similar to LE group (25 µg/mL), therefore, the optimal concentration which was similar to E2 was selected for evaluating the effect of ICI 182,780 (10^-7^) in complementary experiment (Figure 7). As can be seen in the figures, the stimulatory effect of LE and E2 on ALP activity, calcium deposition, and gene expression was declined by ICI 182,780.

## Discussion

As shown in Table 1, Peaks No. 1-9 were identified in licorice extract by comparing with metabolites of *G. glabra* whose structural characteristic, including m/z values, UV spectra, and retention times can be found in the published data (28). Also, further mass fragmentation patterns (HPLC-ESIMS2) in NI mode have to be considered for decisive confirmation of compound structures in the *G. glabra* extract. In previous studies several constituents of the LE such as Hydroxy-4’-O-methylglabridin, hispaglabridin A, and hispaglabridin B were identified as a phytoestrogen and glabridin and glabrene as a phyto-SERM. Components of the extract were in agreement with the previous findings (19, 29 and 30). Hence, the importance of estrogen level in pathogenesis of osteoporosis in women and the effect of E2 in osteoblastogenic differentiation of hBM-MSCs were identified (31), we hypothesized the LE have the potential to prevent osteoporosis through the control over the osteogenic differentiation and proliferation of hBM-MSCs. Moreover, BM-MSCs have recently widespread attention due to their potential use in tissue engineering application and cell therapy; we encourage investigating the effect of LE on differentiation and proliferation of BM-MSC and compared with that of the E2 as a positive control. The results of our experiment demonstrated that the LE has stimulatory effects on proliferation and osteogenic differentiation of mesenchymal stem cells. The proliferation rate of MSCs is consequential in that involvement of MSCs in bone-forming osteoblast and osteogenesis. Cell proliferation results indicate that LE was able to accelerate MSCs proliferation in a dose and time dependent manner (32). Our findings were in line with previous studies showing a stimulatory effect of phytoestrogens, genistein and diadzein, on the proliferation and differentiation of osteoblastic bone marrow stromal cell (33). The beneficial effect was accomplished at a concentration of 10-50 µg/mL and reached a peak at 50 µg/mL after 72 h. LE diminished proliferation at concentration of 100 µg/mL and this biphasic pattern may be related to phytoSERM, glabrene, and glabridin, as Hu *et al.*, has noted the biphasic effect of LE on the growth of MCF-7 breast cancer cells (34). We evaluated the effect of LE on differentiation by monitoring the ALP activity and calcium deposition of MSCs. ALP activity is an early-stage biomarker enzyme that has an important role in osteogenic differentiation of MSCs and calcium deposition that is a late marker of osteogenesis (35). In comparison to control a significant increase in ALP activity was observed on day 6 in the presence of 10-25 µg/mL of LE and reached a peak in 25 µg/mL. In addition, LE accelerated calcium deposition on day 12 at concentration of 25 µg/mL. These data were in agreement with previous studies investigating the effect of flavonoids of *Herba epimedii* and petroleum ether extract of *Cissus quadrangularis* on MSCs (3, 36). Park *et al.* reported that a hot-water extract of *Allium hookeri* roots has a stimulatory effect on proliferation, ALP activity, and mineral deposition of osteoblast–like MG-63 cells (37). The changes in ALP activity and deposition of calcium nodule were accompanied by the up-regulation of the osteogenic marker genes. The elevation in the expression of the bone-related genes, including Runx-2, osteocalcin, ALP, and BMP-2 compared to control were observed. Our results are in accordance with those of others who reported that another phytoestrogen, liquiritigenin, has direct stimulatory effects on bone development in cultured MC3T3-E1 osteoblast cell (36, 38). Lin *et al.* indicating that the combination of licorice extract and quercetin increased in the expression of BMP2 mRNA and protein level of the new bone area in cultured mouse calvaria (39). Also Choi *et al.* indicated that glabridin, an isoflavane of licorice, increases osteocalcin secretion and ALP activity in osteoblast MC3T3-E1 (21). Moreover, glabridin and glabrene also increased creatine kinase activity in epiphyseal cartilage and diaphyseal bone in prepubertal female rats (30). In this study, the inductions in osteoblastic differentiation were mediated through an estrogen like action, Not only was the effect of LE on differentiation similar to that observed with E2, but also these effects were diminished by a pure ER antagonist ICI 182, 780. Consistent with our finding, Choi reported that the elevation of ALP activity and collagen synthesis by glabridin in osteoblastic MC3T3-E1 cells was abolished completely by the anti-estrogen tamoxifen (21).

In conclusion, this is the first evidence that LE can increase the proliferation and osteogenic differentiation of BM-MSCs and having the pattern as well as estradiol. Accordingly LE can be a useful candidate for the prevention of osteoporosis in menopausal women. Further studies are required for evaluating the effect of LE and the components on signaling pathways in bone formation *in-vitro* and *in-vivo*. 

*Supplementary File*

The identified compounds in LE and the RP-HPLC chromatogram at 283 nm and TIC chromatogram in NI mode as well as the flow cytometry of CD31, CD44, and CD73 in hBM-MSCs available as Supplementary File.
