# Expression of Concern for Ethyl Acetate Extract of Licorice Root (*Glycyrrhiza glabra*) Enhances Proliferation and Osteogenic Differentiation of Human Bone Marrow Mesenchymal Stem Cells [Iran J Pharm Res.17(3):e124804]

**DOI:** 10.5812/ijpr-165191

**Published:** 2025-08-05

**Authors:** Editor-in-Chief IJPR

**Affiliations:** 1 School of Pharmacy, Shahid Beheshti University of Medical Sciences, Tehran, Iran

The Editors of the Iranian Journal of Pharmaceutical Research (IJPR) have been made aware of concerns regarding the integrity of certain images presented in the article Ethyl Acetate Extract of Licorice Root Enhances Proliferation and Osteogenic Differentiation of Human Bone Marrow Mesenchymal Stem Cells (1). Comments posted on the post-publication peer review platform PubPeer have highlighted potential image duplication or overlap between figures in this article and figures published in other unrelated publications. The concerns include similarities between:

- Paper 1: Molecular Biology Reports (2024), Figure 2D (PMID 38270663) (2)

- Paper 2: Cell Journal (2016), Figure 2A (PMID 27540522) (3)

- Paper 3: International Journal of Organ Transplantation Medicine (2014), Figure 4B (PMID 25013621) (4)

- Paper 4: Iranian Journal of Pharmaceutical Research (2018), Figure 3C (PMID 30127828) (1)

Additionally, a separate PubPeer comment suggests that Figure 3B in this article appears to be identical or highly similar to Figure 3B in another publication by Soleimani et al. (2012) (5).

Given these concerns, the editorial office has initiated an investigation in accordance with the journal’s ethical guidelines and COPE recommendations. While this process is ongoing, readers are advised to interpret the findings of this article with caution (1).

We will provide an update once the investigation has concluded and further information becomes available.

Editorial Office

Iranian Journal of Pharmaceutical Research (IJPR)

Date: 1 Aug 2025
